# Determinants of intention to get tested for STI/HIV among the Surinamese and Antilleans in the Netherlands: results of an online survey

**DOI:** 10.1186/1471-2458-12-961

**Published:** 2012-11-09

**Authors:** Alvin H Westmaas, Gerjo Kok, Pjer Vriens, Hannelore Götz, Jan Hendrik Richardus, Hélène Voeten

**Affiliations:** 1 Municipal Public Health Service Rotterdam, P.O. Box 70032, 3000, LP, Rotterdam, The Netherlands; 2 Maastricht University, Department of Work & Social Psychology, P.O. Box 616, 6200, MD, Maastricht, The Netherlands; 3 Erasmus MC, University Medical Center Rotterdam, Department of Public Health, P.O. Box 2040, 3000, CA, Rotterdam, The Netherlands

**Keywords:** Health Belief Model, STI testing, Surinamese, Dutch-Antilleans, Subjective norms, Culturally relevant

## Abstract

**Background:**

High infection rates of STIs are found among the different ethnic communities living in the Netherlands, especially among the Surinamese and Dutch-Antilleans. Only limited effective interventions that promote STI/HIV testing among these communities are available in the Netherlands. In the present study we identified the determinants of the intention to get tested for STI/HIV of the sexually active Surinamese and Dutch-Antilleans living in the Netherlands. Secondly, this study assesses which determinants should be addressed when promoting STI/HIV testing among these communities.

**Methods:**

In total, 450 Surinamese and 303 Dutch-Antillean respondents were recruited through Dutch Internet panels and group activities. The questionnaire used in the online survey was based on the concepts of the Health Belief Model, the Social Cognitive Theory, and Theory of Planned behavior. To correct for multiple outcome testing, we considered differences as statistically significant at p<.01 for all analyses. For the multivariate linear regression analysis, variables that were significant were entered into the model block-wise.

**Results:**

Health motivation, cues to action, subjective norms, risk behavior, test history, open communication about sexuality, and marital status were important (univariate) predictors of the intention to get tested for STI/HIV for both the Surinamese and Dutch-Antillean respondents. For both the Surinamese and Dutch-Antilleans, subjective norms were the most salient predictor of the intention to get tested in multivariate analyses, explaining 10% and 13% of the variance respectively; subjective norms had a direct influence on the intention for both the Surinamese and the Dutch-Antilleans.

**Conclusions:**

The strong correlation and predictive power of subjective norms on the intention to get tested for STI/HIV, endorses the importance of focusing on community-based intervention rather than focusing on personal determinants, to change the present perceptions and attitudes towards testing. Health promoting programs should be aimed at promoting open communication regarding sexuality and testing. Stimulating each other to get tested frequently could also help achieving the desired behavior.

## Background

High infection rates of STIs are found among the different ethnic communities living in the Netherlands. Especially among the Surinamese and the Dutch-Antilleans, higher positivity rates were found for chlamydia and gonorrhea [1,2]. It is known that early detection and treatment are necessary for effective prevention. However, only limited effective interventions that promote STI/HIV testing among these communities are available in the Netherlands. In order to effectively address the specific needs of these communities, it could be necessary to make health promoting programs either culturally sensitive or culturally-based. Cultural sensitivity could be described as the extent in which the ethnic characteristics, values and beliefs of the target population are incorporated into the design and pathway of delivery of the health promoting program. Culturally-based refers to the extent in which culture and its core values are used as a medium in health promoting programs in order to achieve the desired behavior [3]. For both types of health promoting programs, it is important to have a good understanding of the determinants related to the health behavior.

Research has shown that interventions that used predictors of the desired behavior to select target groups, and to select suitable methods and applications to target these determinants, tend to have the largest effects in changing behavior [4,5].

Due to lack of understanding the determinants related to STI/HIV testing behavior among the Surinamese and Dutch-Antilleans in the Netherlands, the Municipal Public Health Service (MPHS) of Rotterdam started a research project into these determinants. To ensure that the determinants to be found at the end of the research project were the most relevant ones, we decided to divide the research project in three smaller studies. In 2009, we sequentially started with a qualitative focus group study, followed by a quantitative online study, and a qualitative in-depth interview study. The focus group study was conducted to explore the existing perceptions of the communities, whereas the quantitative online study was conducted to quantify the results found in the focus group study. Additionally, we tried to identify the determinants of the intention to get tested for STI/HIV of the Surinamese and Dutch-Antilleans living in the Netherlands by means of social cognitive theories, and assess which determinants need to be addressed when promoting STI/HIV testing. The qualitative in-depth interview study was conducted to get fuller understanding of the underlying beliefs of the results found in the quantitative study. In both of the qualitative studies, which will be presented elsewhere, the PEN-3 model of Airhihenbuwa was used as theoretical base to centralize culture in the research process [6-8]. The quantitative online survey, which is presented here, was primarily based on the Health Belief Model (HBM) [9,10] and constructs of other social cognitive theories. In short, the HBM tries to explain health behavior by focusing on the individuals’ perception of the threat, and the individuals’ perception of neutralizing this threat. The perception of threat is divided in perceived severity of the threat, and the perceived susceptibility to the threat. Furthermore, the HBM distinguishes between one’s perception on the benefits of showing health behavior, and one’s perception of the barriers in showing the health behavior. Together these are the individuals’ perception of neutralizing the threat. Lastly, the model knows cues to actions, which are defined as triggers to stimulate the individual to perform the health behavior.

In the present study, we identified the determinants of the intention to get tested for STI/HIV of the sexually active Surinamese and Antilleans living in the Netherlands. Secondly, this study assesses which determinants should be addressed when promoting STI/HIV testing among these communities.

## Methods

### Recruitment of participants

The participants for this study were recruited through an Internet research agency called *FlyCatcher*, an Internet research agency called *PanelClix*, and by group activities at a migrant organization in Rotterdam, the Netherlands. Both research agencies have an online Internet panel that is representative for the population living in the Netherlands with regards to age, gender, socio-economic status (SES), and ethnicity. Ethnicity was determined based on the definition of the CBS Statistics Netherlands which uses country of birth to define a person as a migrant [11]. Hereby, the CBS Statistics distinguishes between first generation and second generation migrant: a respondent born in Surinam or the Dutch-Antilles is a first generation Surinamese or Dutch-Antillean. If the respondent was born in the Netherlands and at least one of the parents was born in Surinam or the Dutch-Antilles, the respondent was defined as a second generation Surinamese or Dutch-Antillean. In determining the ethnicity, the country of birth of the mother was leading when it was different from that of the father. This was done because in the Latin American culture, a single mother upbringing is very common.

At first, only the research agency *FlyCatcher* was asked to recruit a random sample of 400 Surinamese and 400 Dutch-Antillean respondents for the online survey. However, the research agency was not able to recruit the desired number of respondents, and therefore cooperated with the partner research agency *PanelClix* to disseminate the questionnaire. Together, the research agencies invited 5267 respondents of which 1160 completed the questionnaire leading to a response rate of 22%. Of this sample, 436 Surinamese and 224 Dutch-Antilleans were included in the survey, after correcting for our definition of ethnicity as the research agencies evidently used another definition of ethnicity that remained unclear. Because of the smaller number of Dutch-Antillean respondents, we organized two sessions of group activities at a migrant organization where most of the Dutch-Antilleans were familiar with. However, this organization stated that the Dutch-Antilleans would be more likely to fill in a pen-and-paper questionnaires (PPQ). Therefore, the online questionnaire was converted to a paper version for the group activities. In total, 99 people participated in the group activities of which 97 completed the questionnaire. The questionnaires were entered into a statistical program by hand; 79 questionnaires were from Dutch-Antillean respondents and 14 from Surinamese (4 were from other ethnic communities and were thus excluded). The respondents that were reached through the Dutch Internet panels, received €1,50 in credits for the completion of the questionnaire. The respondents of the PPQ group received €10 compensation to meet travel expenses.

Ethical approval was obtained from the Ethics Committee of the School of Psychology and Neuroscience, Maastricht University.

### Measurements

The questionnaires used in the survey were written in Dutch, and was based on the concepts of the Health Belief Model (HBM) [9,10,12] and other socio-cognitive models. The variables regarding the constructs of the HBM and other socio-cognitive models were all measured on a five-point Likert scale, unless stated otherwise.

### Constructs of the Health Belief Model

• *Perceived susceptibility* was assessed by one item for both STI and HIV: ‘If I have sex without a condom I have *[very small – very large]* chance to contract (an STI – HIV)’. The two items were combined into one scale (r=.87, p<.001).

• *Perceived severity* was measured by the questions: ‘Can you rate how severe you would find it to contract (an STI – HIV)?. The two questions were combined into one scale (r=.55, p<.001).

• *Health motivation* was measured by combining three beliefs regarding the intention to get tested, even when facing barriers (alpha=.86): ‘If people can see me enter the test facility, the chance I would get tested is *[very small-very large]*’, ‘Even if people could start gossiping about me, the chance I would get tested is *[very small-very large]’,* and ‘Even if I am afraid I’m infected, the chance I would get tested is *[very small-very large]’.*

• *Perceived benefits* were measured by two beliefs for both STI and HIV: ‘I would get tested for (STI – HIV), because I could receive better treatment’ and ‘I would get tested for (STI – HIV), because I can prevent infecting someone’. All four questions were combined into one scale (alpha=.93).

• *Perceived barriers* was measured by the six beliefs (alpha=.92): ‘I do not intend to get tested, because (people can start gossiping about me, I am afraid to disgrace my family in the community, people will think I am “dirty”’, people could see me enter the test facility, it costs me a lot of money, I am afraid of the consequences if I am infected)’.

• *Cues to action* were constructed by two items regarding the testing history of the social environment: ‘Do you know people in your direct social environment (family, friends) who are tested for (an STI – HIV) in the past 12 months?’ If the respondent knew someone who got tested for either STI or HIV, they were scored as having cues to action.

• *Intention* was used as a predictor of the actual behavior [5] and was measured by the same question for both an STI and HIV: ‘I intend to get tested (again) for (STI – HIV) in the coming six months’. The questions were combined into one scale (r= .93, p<.001).

### Constructs of other psychosocial models

Additional to the constructs of the HBM, the constructs self efficacy and outcome expectancies of the Social Cognitive Theory [13,14] were used in the questionnaire.

• *Self efficacy*[15] was measured by the four beliefs (alpha=.92): ‘I think I am able to… (find information about how and where I can get tested, visit the testing facility, discuss my sexual behavior with a nurse, make an appointment to get tested)’.

• *Outcome expectancies* regarding the anticipated social responses [9] after testing was measured by seven beliefs: ‘If I would be infected with an STI and people would know, they (would avoid me, would think I am dirty, would not want to be friends with me anymore, would have less respect for me, would feel uncomfortable around me)’; ‘If I would get tested other people would think I have had (unsafe sex, sex with other persons than my own partner)’. The seven beliefs were combined into one scale (alpha=.92).

Questions regarding normative beliefs and emotional outcomes were used in a similar way as applied by Kakoko et al. [12].

• *Subjective norms* were measured by four items: ‘My (parents, family, friends, community) find it important that I have myself tested frequently’. The four items were combined into one scale (alpha=.91).

• *Social support* was measured by four items: ‘My (parents, family, friends, community) would support me when I would get tested’. The four items were combined into one scale (alpha=.85).

• *Emotional outcomes*[16] was measured by four items: ‘If I would get tested and I would be infected, I would feel (embarrassed, disappointed, guilty, scared)’. The same set of items was asked for HIV. The eight items were combined into one scale (alpha=.90).

### Other variables

• *Socio-demographical variables* measured were *gender*, *age, ethnic background, marital status, relationship status, duration of the relationship, the number of partners, sexual preference, the ethnic background of the partner, type and level of education, religion (phrased as ‘I am….’, with the answers categories ‘Christian’, ‘Protestant’, ‘Catholic’, ‘Muslim’, ‘I have another belief’, and ‘I do not have a belief’),* and *importance of religion*. Other variables measured were the most important *reasons for (not) getting tested, knowledge, open communication about sexuality, test history for STI and/or HIV, timing of the previous test*, and *the outcome of the test*.

• *Risk behavior* was not measured directly. As a proxy, it was constructed by using the items regarding reasons for (not) getting tested for STI/HIV. When respondents selected ‘I had unsafe sex’, or ‘I was afraid I was infected’ as reason for having been tested for STI or HIV, or ‘I was afraid of the test result’ as reason for not having been tested for STI or HIV, they were scored as having had risk behavior.

• *Knowledge* was assessed by a set of six statements regarding STI: ‘The contraceptive pill reduces the chance of an STI’, ‘Most STIs disappear by itself’, ‘If you do not have any complaints, you can still have an STI’, ‘There are STIs that can make you infertile’, ‘There are medicines available to cure STIs’, ‘If you wash yourself after having sexual intercourse, you do not have any risk of an STI’. Another set of six statements was asked regarding HIV: ‘You can be completely cured of HIV’, ‘If you have unprotected sex with someone, you can contract HIV’, ‘If you are HIV infected, it is immediately detectable in your blood’, ‘During an HIV test they only draw blood from you’, ‘You cannot contract HIV through anal sex’, ‘If you was yourself after having sexual intercourse, you do not have any risk of HIV’. Respondents could answer these statements with “right”, “wrong”, or “don’t know”. The score for each set was determined based on the number of correct answered statements (range: 0–6). The two sets were combined into one scale (alpha = .77).

• *Open communication* was measured by three beliefs: ‘It is normal to talk openly about sexuality with my (family, friends, community)’. The three beliefs were combined into one scale (alpha=.80).

After the development of the questionnaire, it was pretested among seven Surinamese and five Dutch-Antilleans to see if the questions were understandable and valid. After the pre-test, some of the questions in the questionnaire were redefined for better understanding, such as use of language and removal of time frame of coming 6 months (except for the questions on intention to get tested). Thereafter it was handed over to *FlyCatcher* and its partner for dissemination among their panel of respondents.

### Analysis

Data were analyzed using the statistical program SPSS version 18. Given the large number of variables in the analyses, we adjusted for multiple outcome testing by considering a p-value of <.01 as significant, for all analyses. Because respondents were recruited in two different ways, we first checked for differences between the respondents who filled in the online survey, versus respondents who filled in the pen-and paper questionnaire (PPQ), by comparing mean scores using the Independent Student *T*-test. For both the Surinamese and Dutch-Antillean respondents, correlations with intention to get tested for STI/HIV in the coming six months were calculated, as well as mean scores and standard deviations of the studied variables.

For the multivariate linear regression analysis, variables that were significant were entered into the model block-wise [17]. The first block of variables contained variables that were related to the Health Belief Model. The second block of variables contained constructs of other cognitive behavioral models. The third block consisted of the other variables such as *risk behavior, open communication* and *the socio-demographical variables*. For the latter, some of the variables were recorded for easy interpretation of the results. *Marital status* was dichotomized; ‘unmarried’ versus ‘living together’/‘married’/‘divorced’/‘widow(er)’. *Level of education* was recorded into three scores; higher educated (university and HBO), average educated (pre-vocational, vocational, higher general secondary education, and pre-university education) and lower educated (primary school, domestic science and similar education). *Religion* was also dichotomized by scoring all religions as ‘1’ and no religion as ‘0’. All other variables were entered into the model as they were asked. Some of the variables were left out of the equation, because they were answered by an insufficiently large group of participants (i.e. less than two thirds of the sample). This was the case for the variables *having multiple partners, timing of the previous test, outcome of STI test, outcome of HIV test, duration of relationship*, and *importance of religion*. Additionally, the respondents with a low intention to get tested for STI/HIV were compared with the respondents with a high intention, by using the Independent Student *T*-test. The variable *intention to get tested in the coming six months* was dichotomized by means of a median split, categorizing “certainly not” and “probably not” (scores 1 and 2 on the 1–5 Likert scale) as low intention and all other answers as high intention. For the Surinamese, this resulted in a 59%/41% low/high ratio; for the Dutch-Antilleans this was 51%/49%.

## Results

Table 1 demonstrates that there are differences in means between the online and the pen-and-paper questionnaire (PPQ) group for both the Surinamese and Dutch-Antilleans. We also checked the direction of the correlates between intention and the other variables. We found that age was the only variable that changed direction between the online and PPQ group for both the Surinamese and Dutch-Antilleans. As the correlates of the online and PPQ group were not that different from each other, the groups were merged together for both the Surinamese and the Dutch-Antilleans. The impact of this decision will be discussed in the Discussion section. Because the ratio of the online and PPQ group were skewed for both ethnic groups, and we are interested in the determinants related to the intention of STI/HIV testing rather than the differences between the ethnic groups, these results will not be discussed in detail in this paper.

**Table 1:** Mean difference between respondents of the online and pen-and paper questionnaire (PPQ) group

	**Surinamese**			**Dutch-Antilleans**		
	**Online group (N=436)**	**PPQ group (N=14)**	p-value*	**Online group (N=224)**	**PPQ group (N=79)**	p-value*
Intention	2.16	3.96	<.001	2.16	3.68	<.001
Perceived susceptibility	4.45	4.58	.6	4.50	4.53	.8
Perceived severity	4.75	4.89	.4	4.69	4.87	.003
Health Motivation	3.84	4.39	.07	3.83	4.17	.02
Perceived benefits	4.16	4.01	.6	4.22	4.44	.1
Perceived barriers	4.18	3.92	.4	4.08	4.25	.2
Cues to action	36.2%	92.9%	<.001	42.4%	63.6%	.001
Self efficacy	4.50	4.57	0.7	4.52	4.49	.7
Outcome expectancies	3.11	3.43	.3	3.26	3.26	1
Subjective norms*	2.77	3.62	.02	2.63	3.55	<.001
Social support	4.02	4.06	.9	4.06	4.32	.03
Emotional outcomes	2.98	2.97	1	3.09	3.03	.7
Knowledge	4.64	4.79	.7	4.64	4.58	.7
Risk behavior	22.5%	21.4%	.9	18.8%	39.2%	<.001
Open communication	3.05	3.90	.01	3.22	3.56	.03
Test history (yes)	47%	64.3%	.2	45.1%	79.7%	<.001
Gender (female)	64.7%	50%	.3	61.6%	64.6%	.6
Age	32.00	21.64	<.001	32.14	24.04	<.001
Marital status (married)	45.2%	7.1%	.01	46.0%	19.2%	<.001
Relationship status (yes)	58.7%	57.1%	.9	58%	59.5%	.8
Education (high)	26.8%	0%	.02	27.2%	16.5%	.06
Religious	67%	85.7%	.1	63.4%	93.5%	<.001

* Using the student’s *t*-test.

The means and standard deviations (SD) of the studied variables of cognitive models are presented in Table 2. Both the Surinamese and Dutch-Antilleans showed average mean intention, perceived themselves as highly susceptible for contracting an STI or HIV, and thought that contracting either of these diseases was very severe. Respondents from both groups scored high health motivation, high perceived benefits, but also high perceived barriers for getting tested. For the Surinamese, slightly more than one third of the respondents (38%) knew people who got tested for an STI or HIV; this was 48% for the Dutch-Antilleans. Both the Surinamese and the Dutch-Antilleans showed high self efficacy, were convinced that their social environment (parents, family, friends and other community members) would support them when they would intend to get tested, and reported high negative emotions when they would get tested and would be infected. As for the correlation with intention to get tested, health motivation, cues to action, and subjective norms were positively correlated for both the Surinamese and the Dutch-Antilleans; for the Surinamese self-efficacy was negatively correlated with intention. The correlations between the studied variables can be found in Additional file 1: TableÂ S1.

**Table 2:** The correlations with intention, means, and standard deviations (SD) of the studied variables (all reported r’s are significant at p<.01)

	**Surinamese**			**Dutch-Antilleans**		
	**r**	**Mean**	**SD**	**r**	**Mean**	**SD**
Intention	-	2.2	1.3	-	2.5	1.5
Perceived susceptibility	ns	4.5	0.9	ns	4.5	0.9
Perceived severity	ns	4.8	0.6	ns	4.7	0.5
Health motivation	.15	3.9	1.0	.25	3.9	1.1
Perceived benefits	ns	4.2	1.2	ns	4.3	1.1
Perceived barriers	ns	4.2	1.0	ns	4.1	1.0
Cues to action (yes)	.17	38%	0.5	.24	48%	0.5
Self efficacy	-.14	4.5	0.8	ns	4.5	0.8
Outcome expectancies	ns	3.0	1.0	ns	3.1	1.0
Subjective norms	.34	2.8	1.2	.40	2.9	1.3
Social support	ns	4.0	1.0	ns	4.1	1.0
Emotional outcomes	ns	4.0	1.0	ns	4.1	1.2

### Prediction of intention to get tested for STI/HIV in the coming six months

The intention to get tested for STI/HIV was first predicted through a regression with the variables of the HBM: health motivation and cues to action. For the Surinamese, these variables explained 5% of the intention to get tested for STI/HIV (Table 3). For the Dutch-Antilleans, the same variables led to an explained variance of 10%. The possible influences of the other variables were explored next. For the Surinamese, both self-efficacy and subjective norms should have been entered into the model. However, self-efficacy was left out of the multivariate analysis, because it behaved in an unexpected direction (people with a high self-efficacy had a low intention to get tested). The explained variance of health motivation, cues to action and subjective norms together was 15%. For the Dutch-Antilleans, only subjective norms were entered in the model, increasing the explained variance to 22%. The last block of variables entered in the model led to an explained variance of 20% for the Surinamese; this was 29% for the Dutch-Antilleans. Although the variable age should have been entered into the models for both the Surinamese and Dutch-Antilleans, it was left out of the equations. The reason for this is related to the different recruitment ways (online survey versus pen-and-paper questionnaire, PPQ). After analyzing the correlation between intention and age for the online versus the PPQ group, we found that the correlation changes direction, for the Surinamese as well as the Dutch-Antilleans. We therefore felt it is inappropriate to include age in the regression analyses, the more because we aim to identify determinants of STI/HIV testing that can be changed with an intervention.

**Table 3:** Prediction of intention to get tested for STI/HIV in the upcoming six months from social cognitive variables and other variables (all reported r’s and beta’s are significant at p<.01)

**Variable**	**Surinamese (N=302)**				**Dutch-Antilleans (N=220)**			
	**r**	**Beta**	**Beta**	**Beta**	**r**	**Beta**	**Beta**	**Beta**
Health motivation	.15	.16	ns	ns	.25	ns	ns	ns
Cues to action	.17	ns	ns	ns	.24	.24	.21	ns
**R**^**2**^		**.05**				**0.10**		
Subjective norms	.34		.32	.25	.40		.36	.29
**R**^**2**^			**.15**				**.22**	
Risk Behavior	.21			ns	.25			ns
Test history (yes)	.22			ns	.28			ns
Open communication	.17			ns	.16			ns
Martital status (married)	-.20			ns	-.25			ns
**R**^**2**^				**.20**				**.29**

For both groups, subjective norms were the most salient predictor, and the only variable with a direct positive influence on the intention to get tested for STI/HIV in the coming six months.

### Differences between low and high intenders

Differences between the low and high intenders on the underlying scales of the variables in the multivariate regression analysis for both the Surinamese and Dutch-Antillean respondents are showed in Table 4. For the Surinamese, the subjective norms regarding frequent testing differed significantly on item level between the low and high intenders. The high intenders more often had had a previous test for an STI or HIV, compared with the low intenders. The former group was also significantly more often able to talk openly about sexuality with their family than the latter.

**Table 4:** Differences between low and high intenders on the underlying scales of the variables in the multivariate regression analysis

**Belief**	**Surinamese**			**Dutch-Antilleans**		
	**Low intention**	**High intention**	**p-value**	**Low intention**	**High intention**	**p-value**
Health motivation – if people can see me enter the test facility	3.7	3.9	.22	3.5	4.0	.003
Health motivation – if people can gossip about me	3.7	3.9	.02	3.6	4.2	<.001
Health motivation – if I am afraid of being infected	4.0	4.0	.50	3.9	4.3	.001
Cues to action – knowing someone tested for a STI	13%	22%	.02	15%	40%	<.001
Cues to action – knowing someone tested for HIV	28%	37%	.05	30%	42%	.05
Subjective norms – parents find frequent testing important	2.4	3.3	<.001	2.3	3.4	<.001
Subjective norms – family find frequent testing important	2.2	3.2	<.001	2.2	3.1	<.001
Subjective norms – friends find frequent testing important	2.3	3.2	<.001	2.2	3.3	<.001
Subjective norms – community find frequent testing important	2.6	3.2	<.001	2.7	3.4	<.001
Risk behavior – got tested for STI because I had unsafe sex	28%	38%	.15	27%	34%	.42
Risk behavior – got tested for STI because I had physical complaints	18%	17%	.82	19%	15%	.60
Risk behavior – got tested for STI because I was afraid of being infected	16%	12%	.38	6%	21%	.01
Risk behavior – not got tested for STI because I was afraid of test result	2%	5%	.26	2%	12%	.005
Risk behavior – got tested for HIV because I had unsafe sex	24%	38%	.07	11%	29%	.02
Risk behavior – got tested for HIV because I had physical complaints	3%	5%	.38	4%	7%	.54
Risk behavior – got tested for HIV because I was afraid of being infected	19%	11%	.14	6%	14%	.20
Risk behavior – not got tested for HIV because I was afraid of test result	3%	6%	.25	6%	9%	.51
I was test before for a STI or HIV	41%	58%	.001	42%	67%	<.001
Open communication – normal to talk about sexuality with family	2.4	2.9	.002	2.7	3.1	.05
Open communication – normal to talk about sexuality with friends	3.7	3.9	.12	3.6	4.1	.001
Open communication – normal to talk about sexuality within my community	2.7	3.0	.02	3.0	3.3	.03

For the Dutch-Antilleans, the low intenders showed significant lower motivation to get tested if people could see them enter the test facility, people could start gossiping about them, and if they were afraid of being infected. The low intenders also knew fewer people in their social environment who were tested for STIs. Just like the Surinamese high intenders, the Dutch-Antillean high intenders are surrounded with people who find frequent testing important. Compared to the low intenders, the high intenders significant more often reported as reason for STI testing that they were afraid of being infected with an STI. However, they also reported significantly more often as reason for not getting an STI test that they were afraid of the test result. The high intenders more often had had an STI and/or HIV test before, and significantly more often found it normal to openly talk about sexuality with their friends.

## Discussion

In this study, we identified the determinants of the intention to get tested for STI/HIV in the coming six months among the Surinamese and Dutch-Antillean communities in the Netherlands, and assessed which determinants need to be addressed when promoting STI/HIV testing among these communities. Results showed that the variables health motivation, cues to action, subjective norms, risk behavior, test history, open communication, and marital status were important predictors (univariately) of the intention to get tested for STI/HIV for both the Surinamese and Dutch-Antillean respondents. Subjective norms (whether a respondent thinks his social environment finds it important to get tested frequently) was the most salient predictor of intention to get tested, and explained 10% and 13% of variance for the Surinamese and Dutch-Antilleans respectively. The Surinamese respondents showed higher intention to get tested in the coming six months when they were surrounded with people who find frequent testing important, when they were previously tested for an STI or HIV, and when they found it normal to openly communicate with their family. The Dutch-Antillean respondents showed higher intention to get tested when they felt motivated despite of possible barriers, when they knew people who were tested for an STI, when they were surrounded with people whom find frequent testing important, when they were aware of their risk behavior, when they were previously tested for an STI or HIV, and when they found it normal to openly communicate with their friends about sexuality.

When interpreting the results some limitations must be considered. First of all, the respondents were recruited in two different ways. The majority was recruited through Dutch Internet panels, and a total of 93 Surinamese and Dutch-Antillean respondents were recruited through group activities in which they filled in a paper version of the online questionnaire. Combining the respondents from the different sampling waves could have influenced the results found in the study (Table 1). For the Surinamese, the pen-and-paper questionnaire (PPQ) group is only 3% of the total, and thus its influence on the analyses can be neglected. For the Dutch-Antilleans, *w*hen only looking at the variables of the multivariate model, we find that differences between the online PPQ group were found for all variables in the model except for *health motivation* and *open communication*. It is possible that the higher perceived severity, higher motivation, and lower mean age of the PPQ group led to a higher mean intention of this group as they are more likely to perform sexual health behavior. However, it is also possible that the higher education of the online panel led to a more realistic perception regarding the threat of STIs, resulting in lower intention. Although the underlying mechanism remains unclear, we should take the possibility of bias into account [18]. A second limitation is that both the Surinamese and Dutch-Antillean communities consist of multiple smaller ethnic communities. For example, the Surinamese community includes Hindustani, Chinese, Creoles, and many other smaller ethnic communities. During the study, the respondents were asked to fill in the country of birth, which made it impossible to discriminate between the smaller ethnic communities during the research. Therefore, the results of the study may not be applicable to these smaller ethnic communities in the Surinamese and Dutch-Antillean community.

Another point of consideration is that we measured a proxy of sexual risk behavior rather than the actual sexual risk behavior, because we felt that questioning the respondents directly on this intimate subject might have led to a dropout of respondents. We believe that the constructed variable is a reasonable proxy for risk behavior, because most respondents would only be afraid of being infected, or afraid of the test results, if they actually had had unsafe sex. However, the constructed variable of sexual risk behavior may have included respondents who perceive themselves as having been at risk while their actual risk was minimal, also known as the ‘worried well’ [19]. Lastly, social desirability bias should be taken into account. Social desirability bias refers to the tendency of respondents to answer questions with responses they believe are socially desired, rather than answering questions by responses which reflect their actual thoughts or feelings [20]. This phenomenon is not uncommon in social studies regarding widely accepted social norms or attitudes, and often occurs when the respondents feel that their answers could be linked back to them. Within the Afro-Caribbean community, it is still perceived as a taboo to talk about sexuality. Although we used an (internet) survey method, which should increase the perceived feeling of privacy among the respondents and therefore lower the social desirability bias, it is still possible that the respondents answered the questionnaire as they felt it should be answered. Also, the perceived prejudices of this community about their sexual behavior could have prevented them to truthfully fill in the questionnaire in order to prevent meeting the beliefs of the social environment. Despite these limitations, we feel that our study provides insight into the determinants related to the intention to get tested among the Surinamese and Dutch-Antilleans, and contributes to the identification of determinants that should be targeted in an intervention.

We found that adding subjective norms to the multivariate regression analysis increased the explained variance for the Surinamese and Dutch-Antilleans with respectively 10% and 13%. This indicates that the intention to get tested for STI/HIV is primarily driven by the approval of the social environment regarding frequent testing, making the subjective norms important predictors. These findings are in contradiction with the meta-analysis of Armitage et al., who found that the subjective norms were the weakest predictor of intention to condom use [21]. However, in the same study it is stated that multiple-item measures of social norms and normative beliefs, like we used in our study, had significant higher correlations with intention than the other measures [21].

Our study also shows that for the Surinamese self-efficacy is negatively correlated with the intention to get tested in the coming six months; the more people perceive themselves as being capable to get tested, the less intention these people show; the same was found for perceived severity. We found some evidence in the data that could help explain the negative correlations found. We found that most (63%) of the Surinamese and Dutch-Antillean respondents with a high intention and low self-efficacy, perceived higher barriers regarding testing than their peers with a low intention and high self efficacy. This finding indicates that we are probably dealing with ‘temporal construal’ [22]; the respondents with a high intention are thinking the behavior through in more detail, because getting tested is relevant for them. However, because these people are analyzing the behavior in detail, they perceive more barriers and show low self efficacy [23]. People who showed high self-efficacy and low intention could be people for whom getting tested is not that relevant. These people could then perceive getting tested as easy, because it does not apply to them. In this case, the negative correlation between self-efficacy and the intention to get tested is caused by the intention, and not by self-efficacy. Therefore, it would be inappropriate to enclose self-efficacy in the model with variables which do predict (i.e. cause) the intention. Secondly, bivariate analysis showed that people with high self-efficacy also perceived higher severity. The people with higher severity perceived higher barriers, and higher emotional outcomes when being infected after testing. These barriers and emotional outcomes could lead to a decrease of their capability to perform the behavior, which could increase denial and defensive reaction towards testing, causing a low intention [24]. The results of the bivariate analysis also indicate that people are afraid of gossiping, and consequently stigmatization, when getting tested. It also indicates that people are afraid of getting a positive test result when getting tested. The consequences of being infected, and therefore stigmatized, could be a reason for a decrease in the intention to get tested. This is similar to the results found in a qualitative study regarding the fear of stigmatization as a barrier to HIV voluntary counseling and testing among South Africans [25].

For the Dutch-Antillean high intenders, almost twice the number of respondents reported to know someone in their direct social environment who got tested for an STI as compared to the low intenders. The Surinamese and Dutch-Antillean high intenders also found themselves more often in a social environment that perceived it as normal to openly discuss sexuality, and among people who found that frequent testing is important. These findings suggest that it is important to focus on these determinants when stimulating STI/HIV testing among the Surinamese and the Dutch-Antilleans. It is expected that when people would discuss sexuality more openly, more people would know others who got tested, which could increase their own intention to also get a test. However, in order to achieve this, both personal norms and subjective norms should be targeted. A possible solution could be found in interventions based on the social norms approach (SNA) [26]. This theory assumes that our behavior is influenced by the perceptions of the social environment on how to behave, and was used in the promotion of safer drinking [27], the prevention of sexual assault [28], safe driving, and smoking behavior [29]. SNA distinguishes three target audiences for an intervention: a whole community including those who are not at-risk (universal), members of a group at-risk (selective), and individuals at-risk. In terms of our study, it could be a good idea to start focusing on the universal audience. By inviting the whole Surinamese and Dutch-Antillean community in the Netherlands to get an STI/HIV test, for example yearly, stigmatization will be lower because no one can see whether or not you have had unprotected sex. People could simply state that they take up the invitation that they received from the testing facility. A similar invitation was sent to youngsters for the participation in a chlamydia screening project in the Netherland regardless whether these youngsters were sexually active or not [2,30]. Over time, testing will become a social norm. Secondly, the individuals at-risk should be targeted by providing them with accurate information on the importance of testing, normative feedback, and coping strategies to promote the desired behavior. By targeting these points-of-entry, targeting both the personal perceptions of individuals and the (social) environmental factors, a future intervention is more likely to effectively promote testing behavior.

## Conclusions

This study provides relevant and important insights for health policy makers who want to improve the STI/HIV testing behavior among the Surinamese and Dutch-Antilleans in the Netherlands. The strong and direct positive, association between subjective norms and the intention to get tested for STI/HIV, endorses the importance of focusing on community-based intervention rather than focusing on personal determinants to change the present perceptions and attitudes towards testing. Other studies confirm that interventions with multiple points-of-entry can successfully promote healthy behavior. Health promoting programs should help these communities to change the social norms in order to achieve the desired behavior. The key message seems to be open communication about sexuality to make it something normal. Further in-depth research will be initiated to get more insights in how an intervention to promote STI/HIV testing among the Surinamese and Dutch-Antilleans should look like, and whether the findings of the present study are applicable to other migrant communities living in the Netherlands.

## Competing interests

The authors declare that they have no competing interests.

## Authors’ contributions

AHW participated in the design of the study, carried out the study, performed the analyses and drafted the manuscript. GK discussed interpretation of results and helped draft the manuscript. PV helped conceiving the study, participated in the design of the study and provided comments on the manuscript. HG discussed interpretation of the results and provided comments on the manuscript. JHR discussed interpretation of the results and provided comments on the manuscript. HV discussed interpretation of results, helped draft the manuscript and coordinated the study. All authors read and approved of the final manuscript.

## Pre-publication history

The pre-publication history for this paper can be accessed here:

http://www.biomedcentral.com/1471-2458/12/961/prepub

## Supplementary Material

Additional file 1: Table S1 Correlations between study variables, means, and SDs, for the Surinamese (N=450); *p<.05, **p<.01. Click here for file
