# Supplementary material for: Determinants of intention to get tested for STI/HIV among the Surinamese and Antilleans in the Netherlands: results of an online survey
Source: BMC Public Health. 2012 Nov 9;12:961. doi: 10.1186/1471-2458-12-961 (PMC3599572; doi:10.1186/1471-2458-12-961)
Supplement: Additional file 1: Table S1 — Correlations between study variables, means, and SDs, for the Surinamese (N=450); *p<.05, **p<.01. [file 1471-2458-12-961-S1.docx]

| Correlations between study variables, means, and SDs, for the Surinamese (N=450; *p<.05, **p<.01 | | | | | | | | | | | | | | | | | | | | | | |
| --- | --- | --- | --- | --- | --- | --- | --- | --- | --- | --- | --- | --- | --- | --- | --- | --- | --- | --- | --- | --- | --- | --- |
|  | Int | Perc. sus | Perc. sev | HeMo | Perc. ben | Perc. barr | CTA | SE | OE | SN | SS | EO | Know | RB | OC | TH | Gen | Age | MS | RS | Edu | Rel |
| Intention | X |  |  |  |  |  |  |  |  |  |  |  |  |  |  |  |  |  |  |  |  |  |
| Perceived susceptibility | ns | X |  |  |  |  |  |  |  |  |  |  |  |  |  |  |  |  |  |  |  |  |
| Perceived severity | -.1* | .21** | X |  |  |  |  |  |  |  |  |  |  |  |  |  |  |  |  |  |  |  |
| Health Motivation | .15** | .15** | ns | X |  |  |  |  |  |  |  |  |  |  |  |  |  |  |  |  |  |  |
| Perceived benefits | ns | .12* | ns | .34** | X |  |  |  |  |  |  |  |  |  |  |  |  |  |  |  |  |  |
| Perceived barriers | ns | .12* | .13** | .26** | ns | X |  |  |  |  |  |  |  |  |  |  |  |  |  |  |  |  |
| Cues to action | .17** | ns | -.13** | .16** | .13** | ns | X |  |  |  |  |  |  |  |  |  |  |  |  |  |  |  |
| Self efficacy | -.14** | .17** | .11* | .23** | .24** | .33** | .14** | X |  |  |  |  |  |  |  |  |  |  |  |  |  |  |
| Outcome expectancies | ns | ns | ns | ns | .11** | -.39** | ns | ns | X |  |  |  |  |  |  |  |  |  |  |  |  |  |
| Subjective norms (N=307) | .34** | ns | ns | .17** | .17** | ns | .13* | ns | ns | X |  |  |  |  |  |  |  |  |  |  |  |  |
| Social support | ns | ns | .13* | .18* | .15** | .27** | ns | .16** | -.14** | .33** | X |  |  |  |  |  |  |  |  |  |  |  |
| Emotional outcomes | ns | .13** | .17** | .10* | .21** | -.12* | ns | .21** | .41** | ns | ns | X |  |  |  |  |  |  |  |  |  |  |
| Knowledge | ns | .16** | ns | .29** | .23** | .28** | .21** | .46** | ns | ns | ns | .13** | X |  |  |  |  |  |  |  |  |  |

|  | Int | Perc. sus | | Perc. sev | | HeMo | | Perc. ben | | Perc. barr | | CTA | | SE | | OE | | SN | | SS | | EO | | Know | | RB | | OC | | TH | | Gen | | Age | | MS | | RS | | Edu | | Rel | |  |
| --- | --- | --- | --- | --- | --- | --- | --- | --- | --- | --- | --- | --- | --- | --- | --- | --- | --- | --- | --- | --- | --- | --- | --- | --- | --- | --- | --- | --- | --- | --- | --- | --- | --- | --- | --- | --- | --- | --- | --- | --- | --- | --- | --- | --- |
| Risk behavior | .21** | .11* | | -.11* | | .17* | | ns | | ns | | .28** | | ns | | ns | | .13* | | ns | | ns | | .19** | | X | |  | |  | |  | |  | |  | |  | |  | |  | |  |
| Open communication | .17** | ns | | ns | | .11* | | ns | | .2** | | .11* | | .17** | | -.24** | | .39** | | .29** | | ns | | ns | | .13** | | X | |  | |  | |  | |  | |  | |  | |  | |  |
| Test history (yes) | .22** | .13** | | ns | | .23** | | .14** | | .19** | | .30** | | .17** | | ns | | .29** | | .11* | | .1* | | .28** | | .48* | | -.16** | | X | |  | |  | |  | |  | |  | |  | |  |
| Gender (female) | ns | ns | | ns | | ns | | ns | | ns | | ns | | ns | | ns | | ns | | ns | | ns | | ns | | ns | | ns | | ns | | X | |  | |  | |  | |  | |  | |  |
| Age | -.22** | | ns | | ns | | ns | | -.17** | | .23** | | -.16** | | ns | | ns | | ns | | ns | | -.14** | | ns | | ns | | ns | | ns | | ns | | X | |  | |  | |  | |  | |
| Marital status (married) | -.20** | | ns | | ns | | -.12* | | -.1* | | .11* | | -.13* | | ns | | ns | | ns | | ns | | ns | | ns | | ns | | ns | | ns | | ns | | .47** | | X | |  | |  | |  | |
| Relationship status (yes) | ns | ns | | ns | | ns | | ns | | ns | | ns | | ns | | ns | | ns | | ns | | ns | | ns | | ns | | ns | | .12* | | ns | | ns | | .39** | | X | |  | |  | |  |
| Education (low=0, high=1) | ns | ns | | ns | | ns | | ns | | ns | | ns | | .12* | | ns | | ns | | ns | | ns | | .18** | | ns | | -.1* | | ns | | ns | | .2** | | .17** | | ns | | X | |  | |  |
| Religious (yes) | ns | ns | | ns | | ns | | ns | | ns | | ns | | ns | | .10* | | ns | | ns | | ns | | -.1* | | ns | | -.1* | | ns | | .13** | | ns | | ns | | ns | | ns | | X | |  |
| Mean | 2.2 | 4.5 | | 4.8 | | 3.9 | | 4.2 | | 4.2 | | 38% | | 4.5 | | 3.0 | | 2.8 | | 4.0 | | 4.0 | | 4.6 | | 22% | | 3.1 | | 48% | | 64% | | 31.7 | | 44% | | 59% | | 26% | | 68% | |  |
| Range | 1-5 | 1-5 | | 1-5 | | 1-5 | | 1-5 | | 1-5 | | 0-1 | | 1-5 | | 1-5 | | 1-5 | | 1-5 | | 1-5 | | 0-6 | | 0-1 | | 1-5 | | 0-1 | | 0-1 | | 13-73 | | 0-1 | | 0-1 | | 0-1 | | 0-1 | |  |
| SD | 1.3 | 0.9 | | 0.6 | | 1.0 | | 1.2 | | 1.0 | | 0.5 | | 0.8 | | 1.0 | | 1.2 | | 1.0 | | 1.0 | | 1.4 | | 0.4 | | 1.1 | | 0.5 | | 0.5 | | 11.8 | | 0.5 | | 0.5 | | 0.4 | | 0.5 | |  |

| Means, SDs, and correlations between study variables for the Antilleans (N=303; *p<.05, **p<.01 | | | | | | | | | | | | | | | | | | | | | | |
| --- | --- | --- | --- | --- | --- | --- | --- | --- | --- | --- | --- | --- | --- | --- | --- | --- | --- | --- | --- | --- | --- | --- |
|  | Int | Perc. sus | Perc. sev | HeMo | Perc. ben | Perc. barr | CTA | SE | OE | SN | SS | EO | Know | RB | OC | TH | Gen | Age | MS | RS | Edu | Rel |
| Intention | X |  |  |  |  |  |  |  |  |  |  |  |  |  |  |  |  |  |  |  |  |  |
| Perceived susceptibility | ns | X |  |  |  |  |  |  |  |  |  |  |  |  |  |  |  |  |  |  |  |  |
| Perceived severity | ns | .28** | X |  |  |  |  |  |  |  |  |  |  |  |  |  |  |  |  |  |  |  |
| Health Motivation | .25** | .19** | .17** | X |  |  |  |  |  |  |  |  |  |  |  |  |  |  |  |  |  |  |
| Perceived benefits | ns | ns | .15** | .30** | X |  |  |  |  |  |  |  |  |  |  |  |  |  |  |  |  |  |
| Perceived barriers | ns | ns | ns | .30** | .12* | X |  |  |  |  |  |  |  |  |  |  |  |  |  |  |  |  |
| Cues to action | .24** | ns | ns | .18** | ns | .13* | X |  |  |  |  |  |  |  |  |  |  |  |  |  |  |  |
| Self efficacy | ns | ns | ns | .22** | .46** | .31** | .16** | X |  |  |  |  |  |  |  |  |  |  |  |  |  |  |
| Outcome expectancies | ns | ns | .12* | -.20** | ns | -.34** | ns | ns | X |  |  |  |  |  |  |  |  |  |  |  |  |  |
| Subjective norms(N=234) | .40** | ns | ns | .19** | .13* | ns | ns | .13* | .17** | X |  |  |  |  |  |  |  |  |  |  |  |  |
| Social support | ns | ns | ns | .18** | .15* | .21** | ns | .26** | -.20** | .27** | X |  |  |  |  |  |  |  |  |  |  |  |
| Emotional outcomes | ns | ns | .23** | ns | .23** | ns | ns | .23** | .35** | .23** | ns | X |  |  |  |  |  |  |  |  |  |  |
| Knowledge | ns | ns | ns | .28** | .36** | .23** | .22** | .43** | -.15** | -.13* | ns | ns | X |  |  |  |  |  |  |  |  |  |

|  | Int | Perc. sus | Perc. sev | HeMo | Perc. ben | Perc. barr | CTA | SE | OE | SN | SS | EO | Know | RB | OC | TH | Gen | Age | MS | RS | Edu | Rel |
| --- | --- | --- | --- | --- | --- | --- | --- | --- | --- | --- | --- | --- | --- | --- | --- | --- | --- | --- | --- | --- | --- | --- |
| Risk behavior | .25** | ns | ns | ns | ns | .12* | .22** | ns | ns | .13* | ns | ns | .11* | X |  |  |  |  |  |  |  |  |
| Open communication | .16** | ns | ns | ns | ns | ns | ns | .24** | ns | .24** | .18** | ns | ns | ns | X |  |  |  |  |  |  |  |
| Test history (yes) | .28** | ns | ns | .31** | .13* | .26** | .48** | .20** | ns | .18** | ns | ns | .21** | .40** | .16** | X |  |  |  |  |  |  |
| Gender (female) | ns | .19** | ns | .25** | .18** | ns | ns | .24** | ns | .24** | .18** | ns | ns | ns | ns | .12* | X |  |  |  |  |  |
| Age | -.26** | .12* | ns | ns | ns | ns | ns | ns | ns | ns | ns | .16** | .12* | ns | ns | ns | ns | X |  |  |  |  |
| Marital status (married) | -.25** | .12* | ns | ns | ns | ns | ns | ns | ns | ns | ns | ns | ns | ns | ns | Ns | ns | .42** | X |  |  |  |
| Relationship status (yes) | ns | ns | ns | ns | ns | ns | ns | -.18** | ns | ns | ns | ns | ns | -.17** | ns | .15* | Ns | ns | .32** | X |  |  |
| Education (low=0, high=1) | ns | -.12* | -.17** | ns | ns | ns | ns | ns | ns | ns | ns | ns | -.19** | ns | ns | ns | ns | .18** | .16** | ns | X |  |
| Religious (yes) | .13* | .13* | .22** | .14* | ns | ns | ns | ns | .12* | ns | ns | ns | .19** | ns | ns | ns | ns | ns | ns | ns | ns | X |
| Mean | 2.5 | 4.5 | 4.7 | 3.9 | 4.3 | 4.1 | 48% | 4.5 | 3.1 | 2.9 | 4.1 | 3.0 | 4.6 | 24% | 3.3 | 54% | 62% | 30.11 | 39% | 59% | 24% | 71% |
| Range | 1-5 | 1-5 | 1-5 | 1-5 | 1-5 | 1-5 | 0-1 | 1-5 | 1-5 | 1-5 | 1-5 | 1-5 | 0-6 | 0-1 | 1-5 | 0-1 | 0-1 | 15-77 | 0-1 | 0-1 | 0-1 | 0-1 |
| SD | 1.5 | 0.9 | 0.5 | 1.1 | 1.1 | 1.0 | 0.5 | 0.8 | 1.0 | 1.3 | 1.0 | 1.2 | 1.3 | 0.4 | 1.1 | 0.5 | 0.5 | 10.5 | 0.5 | 0.5 | 0.4 | 0.5 |
